# Bridging the intervention-implementation gap in primary health care delivery: the critical role of integrated implementation research

**DOI:** 10.1186/s12913-017-2663-8

**Published:** 2017-12-21

**Authors:** John Koku Awoonor-Williams, Ebenezer Appiah-Denkyira

**Affiliations:** Ghana Health Service, Private Mail Bag, Ministries Post Office, Accra, Ghana

**Keywords:** Africa, Health systems strengthening, Primary health care, Maternal, newborn, and child health, Ghana, Mozambique, Rwanda, Tanzania, Zambia

## Abstract

For national and local leaders to achieve universal health coverage, a new approach or technique to gathering evidence and understanding the contexts that influence the outcome of a study and goes beyond the quantitative results of clinical trials and pilot projects is important. The Doris Duke Charitable Foundation’s African Health Initiative (AHI) was designed to produce this type of knowledge through embedding implementation research into Population Health Implementation and Training (PHIT) partnership projects in five countries (Ghana, Mozambique, Rwanda, Tanzania, and Zambia) with the goal of improving primary health care and population health. In Ghana, this integration of research into implementation has contributed to the successful testing, adaptation and implementation of the Community-based Health Planning and Services (CHPS) model (The Navrongo Pilot Project), with results from the AHI-funded work informing national scale-up of effective practices. Further application of implementation science methods and frameworks to study cross-project lessons also produced the evidence needed by national and local decision makers on how and why different intervention components were successful and where and how local context drove implementation and adaptation. Cross-project research also identified effective approaches across diverse settings for building capacity for data-driven improvement, coaching and mentoring clinicians and researchers, developing locally appropriate interventions to reduce neonatal mortality, and integrating implementation research to inform local implementers and researchers in more effective strategies to strengthen health systems and improve health services and population health. Evidence has already shown the potential for this type of work to accelerate regional learning and spread of successful interventions to achieve targeted health goals more efficiently, better enabling countries to achieve the ambitious, but important, U.N. Sustainable Development Goals.

## Background

The last century has seen remarkable advances in the knowledge of how to better prevent and treat many of the conditions that are major causes of suffering and death around the world. The U.N. Millennium Development Goals helped spur considerable progress in reducing deaths caused by infectious diseases such as HIV, malaria and TB, as well as in addressing major causes of maternal and child deaths [1]. Efforts to strengthen primary health care in low- and middle-income countries have contributed to these gains, by improving access to needed care among the most vulnerable [2]. However, this success has been tempered by variability in progress between and within countries, lack of global access to effective primary care, the rise in non-communicable diseases and the chasm between what interventions we know work and what services people actually receive [3, 4]. Due to a decrease in resources available to fund health care delivery, particularly for low and middle income countries, more effective and efficient implementation of interventions is required. In addition, while strong primary care delivery systems can serve as effective platforms for further health system strengthening initiatives, the recent Ebola outbreak made apparent the weaknesses of health systems that are not resilient and people-centered [5–8].

For national and local leaders to continue making progress while addressing these challenges, a new type of evidence is needed that goes beyond the results of clinical trials and pilot projects. Green and Glasgow wrote, “if we want more evidence-based practice we need more practice-based evidence” [9]. This approach increases the probability that evidence from research on effective interventions is useful, relevant and able to inform policies needed to translate this knowledge into action, reducing the chasm between what works and what is delivered, a major cause of suffering and waste globally [10, 11].

### Better knowledge to drive quality universal health coverage

The articles in this supplement of BMC Health Services Research offer a unique opportunity to learn from the African Health Initiative (AHI) funded projects, which focused on strengthening the primary health care delivery systems at the district or provincial levels in five sub-Saharan African countries to improve population health and serve as platforms for further health system strengthening work [12]. Teams from across the projects, representing a range of stakeholders and disciplines, have applied implementation research methodologies to extract learnings from common intervention components and implementation approaches. This type of evidence serves as the bridge between the current know-do gap and is core to the development of resilient people-centered health systems that deliver quality universal health coverage. The articles in this supplement are part of a growing body of implementation research that has increased the knowledge base on how to better adapt and implement interventions effectively across a range of contexts and to cull generalizable lessons from the implementation of interventions developed and tested in other settings [13, 14]. Parry and colleagues argue that the study of health care improvement initiatives needs to answer not simply if an intervention worked, but also apply a theory-driven evaluation approach to provide evidence on how and in what contexts it worked and where change was needed [15]. The supplement authors’ use of mixed methods and implementation research frameworks such as the Consolidated Framework for Implementation Research, is a step towards generating rigorous and more actionable knowledge to strengthen health systems in the five African contexts and to promulgate the translation of knowledge into practice [16–18].

### Better measurement, better improvement

The cross-site research discussed in this supplement reveals effective methods to improve utilization of routine data for improved decision-making. Globally, there is recognition that while data collection and reporting has increased, a gap remains between quality and availability of health information and use by providers and program managers to identify gaps and drive improvement at the clinical and systems level [19–22]. The AHI projects utilized various approaches in the gathering and analysis of health information to inform policy across diverse settings, which helped to improve the availability and quality of data for ascertaining the successful implementation of the interventions amidst changing the culture and capacity for the improvement and use of data for decision making [16–18, 23, 24]. These lessons are important for both policymakers choosing how and where to effectively invest in routine Health Information Systems (HIS), and for HIS users attempting to bridge the gap between data collection and data use to improve the delivery of quality health services [25].

### Better investment in people

The AHI projects were also unique in emphasizing the integration of implementation research into the frontlines, and building capacity of implementers and local researchers within the countries to drive this work during the project periods and for the future [26]. Locally designed and driven research is a key approach to ensuring that health services and delivery processes are more relevant to local challenges and also increase ownership and drive translation from research into practice. The emphasis on implementation research enabled researchers to better understand outcomes and impact, as well as how and why different interventions worked and where variability could inform future adaptation and spread [23, 24]. Further investment in people was seen in the mentoring work focused on increasing capacity in clinicians and managers to be more effective and in strengthening data utilization capacity in targeted data consumers [23, 24].

### The Ghana perspective

In Ghana, this integration of research into implementation has led to the successful testing, adaptation and implementation of the Community-based Health Planning and Services (CHPS) model (The Navrongo Pilot Project), with results from the AHI-funded work informing national scale-up of effective practices. These included the successful implementation and impact of the Ghana Essential Health Intervention Program (GEHIP) on under-five mortality rates and improvements in facility-based maternal and neonatal survival among emergency cases through innovations in improving the referral service [27]. Further changes in the Ghanaian health system, including the National Health Insurance Scheme and decentralization of responsibility and authority, offer current and future opportunities for ongoing integrated research and application of effective interventions. Our experiences have also been informed by and contributed to the broader learning across the AHI-funded projects in areas from neonatal mortality reduction to improving data utilization to drive improvement, research capacity building, and the role of mentoring and coaching to improve quality of care.

## Conclusion

This supplement is an example of applying these emerging methodologies across the five AHI projects to produce the evidence needed by national and local decision makers on how and why different intervention components are successful, and equally important insights into where and how local contexts drive implementation and adaptation. Although the implementation research outcomes described in this supplement do not speak to the traditional population health impact metrics as a measure of success, they provide insight into the acceptability, adoption and early evidence of sustainability of interventions, critical information for policy makers and frontline implementers as they work to achieve stronger health systems and better primary health care [28]. The potential for initiatives like AHI to accelerate regional learning and the spread of effective implementation approaches is enormous, and similar efforts are needed to help countries more rapidly achieve the much broader agenda of the U.N. Sustainable Development Goals, particularly Goal #3, which has health systems strengthening at its core.

### Key messages

- Embedded research helps to articulate the evidence associated with two or more sub units of analysis of a phenomenon. Embedded implementation research is effective in producing usable evidence for policy makers and managers able to be translated into strategies and spread at the national and subnational levels
- Embedded research, despite having been found to be useful in understanding the implementation of health interventions, has not had much written about it. At the same time the need for clear research evidence to improve health interventions continues to be a gap and the subject of academic and policy interest. Embedded implementation will fill this knowledge gap by giving an insight into how local context influences the implementation and adaptation of a project.
- Integration of implementation research can build local capacity of implementers and encourage them to be more responsive to scientific evidence which will help them to deliver better health care within a resource-constrained setting.
- Despite differences in the African Health Initiative projects and countries, key lessons in how to build capacity for embedded implementation research, increase data utilization and improve primary health care offer important insights for other countries working on how to achieve quality universal health coverage. Capacity building, coaching and mentoring of implementers in undertaking embedded implementation research is unique in terms of its contribution to helping frontline managers to understand the process and content that increase the success of implementation of a health intervention. In these specific projects, it further helped frontline managers to articulate the outcomes and impact of the intervention and address the inherent challenges of implementation in a timely manner.

## Abbreviations

AHI: African Health Initiative
CHPS: Community-based Health Planning and Services
GEHIP: Ghana Essential Health Intervention Program
PHIT: Population Health Implementation and Training partnerships
